# Supplementary material for: Distinct Features Based on Partitioning of the Endophytic Fungi of Cereals and Other Grasses
Source: Microbiol Spectr. 2023 May 11;11(3):e00611-23. doi: 10.1128/spectrum.00611-23 (PMC10269846; doi:10.1128/spectrum.00611-23)
Supplement: Supplemental file 1 — Supplemental material. Download spectrum.00611-23-s0001.docx, docx file, 0.9 MB [file spectrum.00611-23-s0001.docx]

**Supplementary tables**

| **Table S1.** Geographic location of sampling sites and sample sizes. Numbers at the right and left of the slash indicate numbers of samples yielded from sequencing and used in statistical analysis. | | | | | | | |
| --- | --- | --- | --- | --- | --- | --- | --- |
| Sampling sites | Coordinates | *Aegilops peregrina* | *Aegilops sharonenesis* | *Avena sterilis* | *Hordeum spontaneum* | *Triticum aestivum* | *Triticum dicoccoides* |
| Almagor | N32°54'4.32", E35°36'55.44" | 0 | 0 | 0 | 0 | 26/32 | 34/39 |
| Eliad | N32°47'25.73", E35°44'57.34" | 0 | 0 | 0 | 0 | 39/41 | 35/42 |
| Ramot Menache | N32°36'6.8", E35°4'9.05" | 31 | 0 | 30 | 26/30 | 38/42 | 42/44 |
| Karkur | N32°28'9.23", E34°59'26.59" | 31 | 0 | 31 | 26/28 | 0 | 0 |
| Hadera Elyachin | N32°24'51.44", E34°54'44.78" | 0 | 0 | 0 | 0 | 41/42 | 0 |
| Netanya | N32°24'17.1", E34°52'49.12" | 0 | 43 | 0 | 0 | 0 | 0 |
| Arsuf Gaash | N32°13'17.11", E34°49'13.3" | 0 | 29/35 | 0 | 0 | 37/40 | 0 |
| Palmachim | N31°55'57.29", E34°43'44.15" | 0 | 37/39 | 0 | 0 | 41/43 | 0 |
| Yashresh | N31°54'57.89", E34°50'33.79" | 31 | 0 | 31 | 27/29 | 0 | 0 |
| Carmia | N31°36'10.51", E34°32'48.7" | 0 | 0 | 0 | 0 | 30/38 | 0 |
| Gvar'am | N31°36'8.64", E34°34'34.07" | 31 | 0 | 29/31 | 26/29 | 0 | 0 |
| Zikkim | N31°36'8.17", E34°31'1.49" | 0 | 38/40 | 0 | 0 | 0 | 0 |
| Be'eri | N31°26'11.65", E34°29'37.9" | 28/31 | 0 | 31 | 30 | 0 | 0 |

| **Table S3.** Fungal classes commonly seen in wheat stem endophytes and their relative abundance. | | | | | | |
| --- | --- | --- | --- | --- | --- | --- |
| Country | Ascomycota | | Basidiomycota | | | References |
|  | Doth^*^ | Sord | Agar | Micr | Trem |  |
| Denmark | 23.0 | 0.3 | - | 16.1 | 31.5 | (56) |
| US | 39.7 | 12.9 | 10.7 | 1.7 | 1.9 | (86) |
| Italy | 30 | 7.5 | 2.5 | 4.4 | 30 | (74) |
| Israel | 39.7 | 7.4 | 1.3 | 4.5 | 19.2 | (40) |
| Denmark | 27.8 | 48.1^**^ | - | 8.4 | 13.7 | (57) |
| UK | 8.3 | 14.3 | 0.6 | 32.9 | 9.8 | (75) ^***^ |

^*^ Abbreviations: Doth – Dothideomycetes, Sord – Sordariomycetes, Agar – Agaricomycetes, Micr – Microbotryomycetes, Trem - Tremellomycetes.

^**^ Rojas et al. (57) included *Fusarium* head blight individuals in study, which resulted in a high proportion of Sordariomycetes in dataset.

^***^ Latz et al. (75) dataset contained a large amount of pathogenic *Blumeria graminis* sequences (accounted for 83% of overall reads). The relative abundance in the table was recalculated with removal *B*. *graminis* sequences from the dataset.

**Supplementary figures**


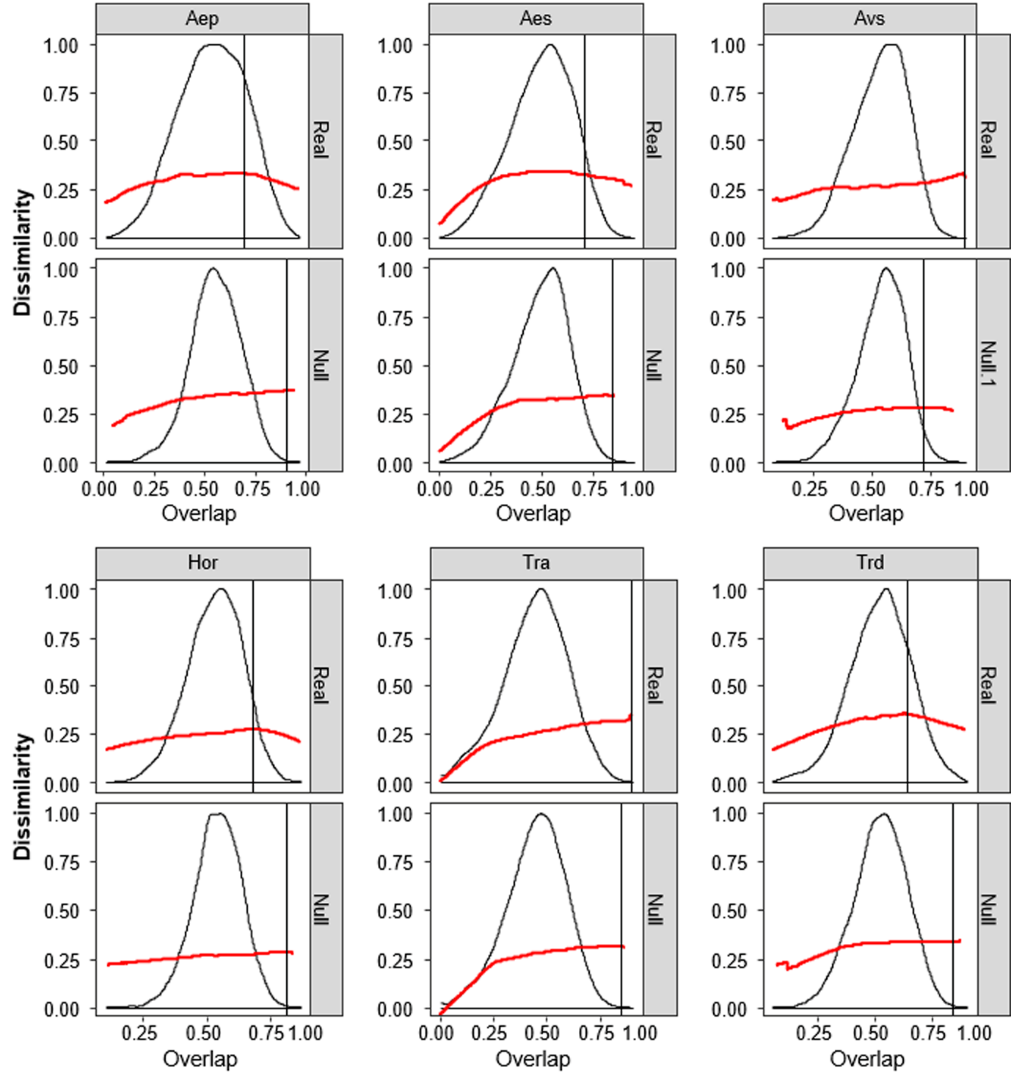


**Figure S1*.*** The DOCs of meta-communities in the six host plants. The overlap distributions of the real and randomized between-subject sample pairs are shown as black curves. The vertical black line represents the change point.

**
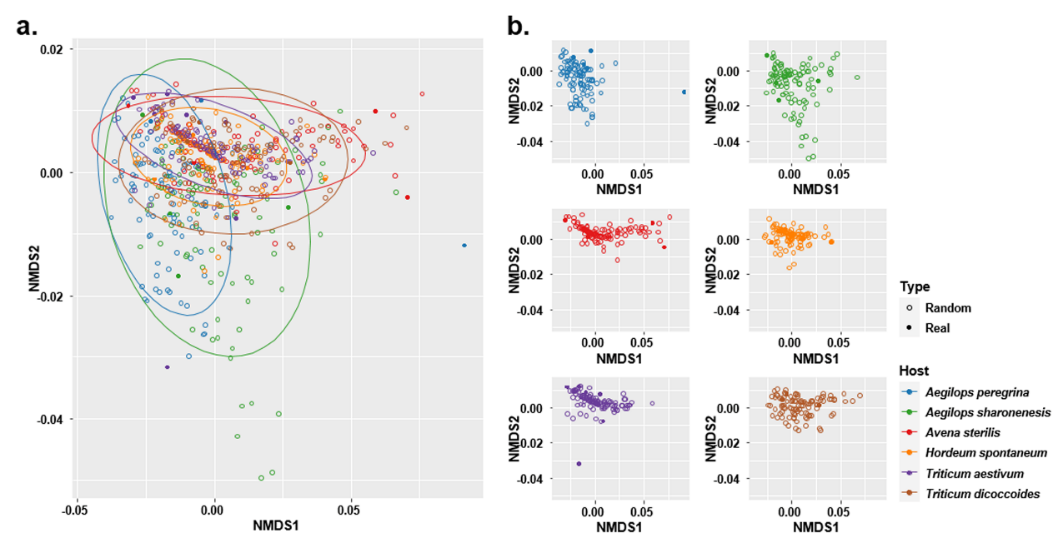
**

**Figure S2.** Comparison of the co-occurrence networks of FECs in the six plant species. **a.** NMDS ordination plots illustrate the dissimilarity between co-occurrence network structures among the hosts. The open circles are the simulated networks, solid circles are networks of real communities from different sites, and ellipses indicate 95% confidence. **b.** A split view of panel **a,** according to the hosts.


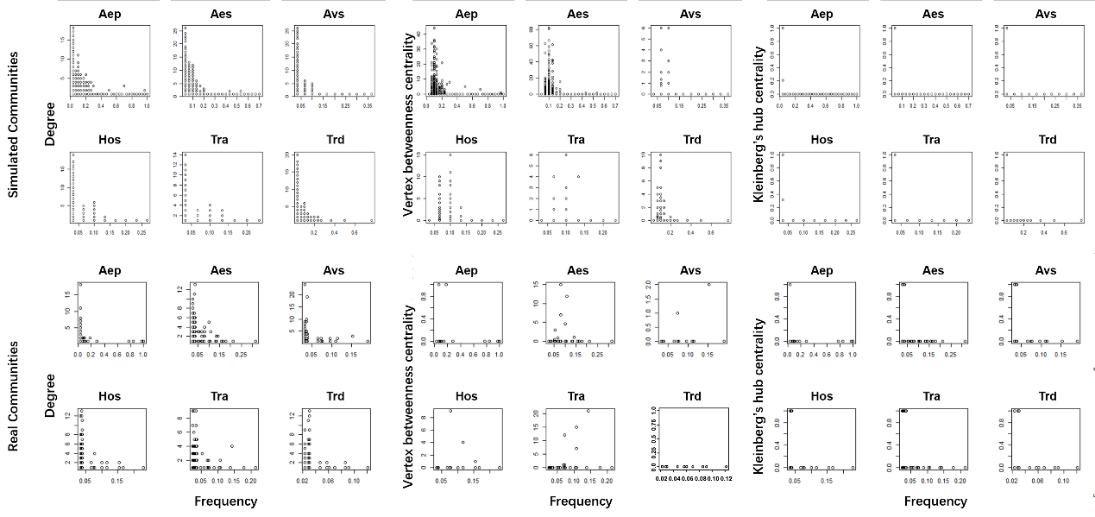


**Figure S3.** Characteristics of co-occurrence networks generated from real and simulated sub-communities. The degree, betweenness centrality, and hub centrality of endophytic fungal taxa in the co-occurrence networks decreased as their frequencies in sub-communities increased. The upper plots were produced from 100 simulated sub-communities, and the lower plots were from real, local communities from different sites. Abbreviations: Aep, *Aegilops peregrina*; Aes, *Aegilops sharonensis*; Avs, *Avena sterilis*; Hos, *Hordeum spontaneum*; Tra, *Triticum aestivum*; Trd, *Triticum dicoccoides*.

**Supplementary materials and methods**

*Data collection*

The data collection strategy was described in the main text.

*Quality control and taxonomy assignment (based on ASVs and OTUs)*

Initially forward and reverse reads were demultiplexed and the quality control check was performed in QIIME2 platform (version 2018.11). The primers were trimmed using the Cutadapt tool (1). Subsequently, the DADA2 workflow (2) was used for quality filtering, dereplication and sample inference. Following quality control, the reverse reads were excluded as they invariably showed poor quality. The 1,032 samples were dereplicated to form unique sequences. The average of the positional qualities from the dereplicated reads was retained as the consensus quality profiles of unique sequences to inform the error model of the subsequent pooled sample inference step, thereby increasing the accuracy of the DADA2 algorithm. This process yielded 3,438 amplicon sequence variants (ASVs), where chimeric amplicons were identified using the “removeBimeradenovo” function and discarded. The demultiplexed pyrosequencing data and associated metadata have been deposited in the Sequence Read Archive, National Center for Biotechnology Information (SRA, NCBI, www.ncbi.nlm.nih.gov/sra) under BioProject ID: PRJNA592195, PRJNA509176.

The fungal taxa were identified based on a workflow combining ASVs and operational taxon units (OTUs). Firstly, the taxonomy of fungal ASVs was assigned using Naïve Bayes approach with minimum 75 bootstrap calls following DADA2 workflow (2) against UNITE general FASTA release for Fungi version 8.0 (3). In preliminary analysis we noticed that the UNITE general FASTA release for Fungi might faile to detect non-fungal ASVs, while the UNITE general FASTA release for eukaryotes incorrectly assigned certain fungal groups (for example, ASVs in a *Cladosporium* clade) to non-fungal ASVs. Therefore, we took the taxonomical assignments from both references into account in the current analysis. The ASVs were searched against the UNITE general FASTA release for Fungi version 8.0 using the Naïve Bayes approach with minimum 75 bootstrap. Non-fungal ASVs were verified by additional tests using the same approach against UNITE general FASTA release for eukaryotes version 8.0, and verified non-fungal ASVs were removed from dataset.

Secondly, for those ASVs in dataset that were not assigned to fungal species, they were clustered into operational taxonomic units (OTUs) based on 97% similarity with function *otu* in “kmer” package (4). One sequence representative within the OTU was identified based on UNITE references with the same approach mentioned above. We then combined the identified ASVs and OTUs to one taxon-sample dataset, transformed the dataset to phyloseq object, and agglomerated the ASVs or OTUs of identical assignments at species level using *tax_glom* function in “phyloseq” package as described in DADA2 workflow (2, 5).

*Core mycobiota definition*

In order to address possible ecological roles of “core” members, we defined the core mycobiota based on prevalence, or “overlapping” patterns of each community member (6, 7). The taxon prevalence amid local communities as well as meta-community were both considered in our study. Therefore, a core member at a given threshold was determined, if its frequency in a local community was above the threshold, meanwhile, which happened more frequent amid meta-community than the threshold. For example, if the threshold was set to 50%, a core taxon should occur on no less than 50% of the samples in a sub-community, which should comply in no less than 50% sub-communities of the meta-community. The definition is represented with following formulas:

*Core’* = {*Tax_i_* | *Occurrence_i_*/*Nsamp* > *Thres*}

*Core* = {*Tax_i_* | n(*Tax_i_*∈*Core’_j_*)/*Ncomm* > *Thres*}

where, *i*, the *i*th taxon; *j*, the *j*th sub-community (or local community) in the meta-community; *Core’*, the taxa with frequency above the given threshold in a sub-community; *Tax_i_*, the *i*th taxon in meta-community; *Occurrence_i_*, times of the *Tax_i_* occurred on samples in a sub-community; *Nsamp*, the number of samples in a sub-community; *Thres*, the threshold assigned; *Core*, the core taxon set; n, cardinal number of (*Tax_i_*∈*Core’_j_*); *Ncomm*, the number of sub-communities in the meta-community.

*Core mycobiota determination*

With regard to studies focused on meta-community which included multiple local communities, *e*.*g*., wheat FECs from different locations with certain environmental heterogeneity, we took local communities as replicates into consideration. The present meta-community was composed by plant individual samples ranged from 111 to 240 due to host identities collected from 3 to 7 sites. Considering the unevenness of samples, we identified the core taxa and tested their robustness with bootstrapping strategy. In present study, 30 samples were randomly selected from sample pool of a certain host to generate a simulated random community. One hundred random communities were generated for on host. Core members were diagnosed with the criteria above for real local communities and 100 simulated sub-communities, respectively. Core sets were determined when real communities and simulated communities yielded same core members. The dissimilarity between the core sets calculated with real and simulated communities at certain threshold were represented with Jaccard Index.

*Co-occurrence network and shared association*

Co-occurrence association among endophytic taxa at fungal species or OTU level were evaluated and Spearman's *rho* were calculated with *cor.test* function in “stats” package (8). The associations were calculated for each sub-community, where the associations with |*rho*| > 0.8 and *p* < 0.001 were remained. In order to explore the consistent co-occurrence associations among sub-communities within a meta-community, the frequencies of each co-occurrence association (*viz*., edges in a co-occurrence network) occurred in co-occurrence networks of each sub-community were recorded and illustrated in a combined network graph. The degree, closeness centrality, and betweenness centrality of each node, and the frequency of edges in the combined network graph were calculated to detect core interactions (7, 9). In addition, 100 simulated communities were also used to calculate shared co-occurrence associations with same method and criteria. The dissimilarities (*d*) between network structural were calculated with methods described in Schieber et al. (10). The *d* between networks was computed considering w1=0.45, w2=0.45 and w3=0.1, and distance matrix was ordinated with NMDS.

**Reference**

1. Martin M. 2011. Cutadapt removes adapter sequences from high-throughput sequencing reads. EMBnetjournal 17:10-12.

2. Callahan BJ, Sankaran K, Fukuyama JA, McMurdie PJ, Holmes SP. 2016. Bioconductor workflow for microbiome data analysis: From raw reads to community analyses. F1000Research 5:1492.

3. Nilsson R, Larsson K-H, Taylor A, Bengtsson-Palme J, Jeppesen T, Schigel D, Kennedy P, Picard K, Glöckner F, Tedersoo L, Saar I, Kõljalg U, Abarenkov K. 2018. The UNITE database for molecular identification of fungi: handling dark taxa and parallel taxonomic classifications. Nucleic Acids Research 47:D259–D264.

4. Wilkinson S. 2018. kmer: an R package for fast alignment-free clustering of biological sequences. <https://cran.r-project.org/package=kmer>. Accessed

5. McMurdie PJ, Holmes S. 2013. phyloseq: An r package for reproducible interactive analysis and graphics of microbiome census data. PLOS ONE 8:e61217.

6. Turnbaugh PJ, Ley RE, Hamady M, Fraser-Liggett CM, Knight R, Gordon JI. 2007. The human microbiome project. Nature 449:804–810.

7. Shade A, Handelsman J. 2012. Beyond the Venn diagram: the hunt for a core microbiome. Environmental Microbiology 14:4-12.

8. R_Core_Team. 2020. R: A Language and Environment for Statistical Computing, *on* R Foundation for Statistical Computing. <https://www.R-project.org/>. Accessed

9. Toju H, Peay KG, Yamamichi M, Narisawa K, Hiruma K, Naito K, Fukuda S, Ushio M, Nakaoka S, Onoda Y, Yoshida K, Schlaeppi K, Bai Y, Sugiura R, Ichihashi Y, Minamisawa K, Kiers ET. 2018. Core microbiomes for sustainable agroecosystems. Nature Plants 4:247–257.

10. Schieber TA, Carpi L, Diaz-Guilera A, Pardalos PM, Masoller C, Ravetti MG. 2017. Quantification of network structural dissimilarities. Nature Communications 8:10.
